# Intergenerational transmission of maternal prenatal anxiety to infant fearfulness: the mediating role of mother-infant bonding

**DOI:** 10.1007/s00737-024-01475-9

**Published:** 2024-06-11

**Authors:** Sofie Rousseau, Danielle Katz, Avital Schussheim, Tahl I. Frenkel

**Affiliations:** 1 Baruch Ivcher School of Psychology, Reichman University, Herzliya, Israel; 2 School of Education, Ariel University, Ariel, Israel; 3 Department of Psychological and Brain Sciences, University of Delaware, Newark, Delaware United States

**Keywords:** Infant negative reactivity, Infant temperamental fearful withdrawal, Maternal-infant bonding, Maternal anxiety, Prospective

## Abstract

**Purpose:**

This study is the first to directly investigate the mechanistic role of maternal bonding toward her infant in the early intergenerational pathway of risk from maternal anxiety to infant fearfulness.

**Methods:**

Mothers (*N* = 216; *M*_age_=32.78) reported on their anxiety and bonding at four time-points between pregnancy and ten-months postpartum. At four and ten-months postpartum, infant temperamental precursors of anxiety were assessed through maternal report and observation.

**Results:**

Cross-lagged longitudinal path modeling indicated a significant link between prenatal maternal anxiety and infant temperamental fearful withdrawal at 10-months postpartum (*R*^2^ = 0.117), which was fully explained by decreased maternal bonding at one-month postpartum and increased infant temperamental negative reactivity at 4-months postpartum.

**Conclusion:**

Results support the need to foster maternal bonding in preventive perinatal care, particularly in the context of maternal anxiety.

## Introduction

Maternal anxiety in the perinatal period is highly prevalent, with meta-analytic work indicating an incidence of approximately 15.0-24.6% for self-reported symptoms and 9.9–15.2% for clinical diagnosis of any anxiety disorder onsetting during the perinatal period (Dennis et al. 2017). Maternal anxiety in the perinatal period may be specific to pregnancy, childbirth, labor or motherhood (e.g. Huiznik et al., 2004; Sjögren 1997; Suchanecki and Goutaudier 2022). Alternatively, perinatal maternal anxiety may be less specific, and rather encompass a wide range of topics – commonly referred to as “general perinatal anxiety” (Dennis et al. 2017; Korja et al. 2017). Maternal anxiety that is specific to pregnancy, childbirth and motherhood, commonly refers to specific worries or concerns that mothers endorse across the perinatal period such as fear of giving birth, negative experiences with the health care system, fear of a potentially traumatic childbirth experience, worries about maternal physical appearance, fear of pregnancy-related change in romantic relationships, and worries pertaining to parenting (Huiznik et al., 2004; Sjögren 1997; Suchanecki and Goutaudier 2022). General perinatal anxiety refers to nonspecific concerns experienced by mothers during the perinatal period, including excessive worry about everyday issues and situations, unrealistic view of problems, general irritability, and the experience of somatic symptoms such as headaches, nausea, and tension (DSM–5; American Psychiatric Association 2013). Importantly, mothers experiencing general perinatal anxiety are likely to also show more specific concerns related to pregnancy, labor, or motherhood and vice versa (Huiznik et al., 2017; Mudra et al. 2020; Rousseau et al. 2021). In the present study, “perinatal maternal anxiety” refers to maternal general anxiety measured during pregnancy and across the first four months postpartum.

Previous research has well documented the long-lasting negative effects of maternal perinatal anxiety on offspring’s development (Rogers et al. 2020 for meta-analysis). Maternal perinatal anxiety has been associated with perturbations in child development from infancy through adolescence, across a wide range of domains (Rogers et al. 2020). Research on maternal anxiety beyond the perinatal period has revealed links between maternal and child anxiety, leading scholars to suggest patterns of intergenerational transmission (Lawrence et al. 2019). Moreover, studies have delineated emerging indicators of intergenerational transmission of anxiety very early on, and within the first year of life. Specifically, literature has revealed links between perinatal maternal anxiety and early infant temperamental dispositions (Davis et al. 2004; Korja et al. 2017) which have been identified as significant precursors of later childhood anxiety. While genetic mechanisms may account for up to 50% of the early intergenerational linkage between mother and child anxiety (Pérez-Edgar and Fox 2018), the importance of early environmental influences has yet to be explored. Studies on older children aged 11–18 years point toward the role of parent-child interaction patterns in mediating links between mother and child anxiety (Waite et al. 2014). Surprisingly, to date no study has examined the extent to which the early parent-infant relationship might underlie the link between perinatal maternal anxiety and early infant temperamental precursors of anxiety. Identification of such early indirect mechanisms would have pivotal clinical value, informing the planning of preventive interventions and allowing for the modifying of risk pathways as early as pregnancy, and prior to the onset of childhood anxiety.

## Infant temperamental precursors of anxiety

Temperamental tendencies of fearful withdrawal are thought to play a central role in the pathogenesis of later childhood anxiety (Sandstrom et al. 2020). Temperamental fearful-withdrawal refers to infants’ dispositional tendency to experience high distress, worry, and fear, while behaviorally displaying withdrawal when facing unfamiliar situations, objects, and people (Gartstein and Rothbart 2003). Tendencies for fearful withdrawal can be seen as early as the first year of life (Braungart-Rieker et al. 2010; Hane et al. 2008) and may intensify over time leading to distinct behavioral inhibition (BI) in early toddlerhood. BI is a broad and persistent response style to novel stimuli that is typified by fearful, inhibited, and distressed behavior along with severe shyness and social avoidance in toddlerhood and early childhood (Degnan and Fox 2007; Kagan 1997; Pérez-Edgar and Fox 2018). Significant BI is seen in approximately 15-20% of children (e.g., Clauss and Blackford 2012) with distinct behavioral indices reliably observed within the laboratory from the beginning of the second year onward (Chronis-Tuscano et al. 2009; Fox et al. 2015). Research establishes BI as the most prominent marker for later anxiety across the lifespan: during childhood (White et al. 2017), adolescence (Chronis-Tuscano et al. 2009) and adulthood (e.g., Frenkel et al. 2015; Moffitt et al. 2007). Taken together, meta-analytic work indicates that infant fearful withdrawal increases the odds of associated BI and subsequent anxiety by almost threefold (Sandstrom et al. 2020). Given such evidenced stability and persistence of fearful withdrawal tendencies, and its associated risk for later BI and anxiety, empirical effort has been invested in identifying early behavioural risk markers that can be observed prior to the distinct expression of fearful withdrawal.

Temperamental Negative Reactivity (NR) has been identified as an early precursor of fearful withdrawal, observable as early as four months of age (Calkins et al. 1996; Fox et al. 2001, 2015). Temperamental reactivity refers to dispositional, biologically driven (Fox et al. 2001), individual differences in both biological and behavioral responses to the environment (Rothbart and Bates 2006). Temperamental NR is typified by high motor activity (e.g., arm waves, leg kicks) and negative affective response (e.g., fuss, cry) in response to both novel social and non-social stimuli (Calkins et al. 1996; Fox et al. 2001). Studies conducted across several cohorts reveal that approximately 50% of NR babies go on to display temperamental fearful avoidant tendencies in early toddlerhood (Fox et al. 2001; Kerr et al. 1994). In sum, the above work highlights a clear developmental pathway of risk that unfolds across the first year of life, starting from early temperamental NR, identifiable in the first months of life, culminating into persistent fearful withdrawal by the end of the first year of life.

### Links between perinatal maternal anxiety and early infant temperamental precursors of anxiety

Anxiety aggregates in families, with empirical data indicating clear links between both clinical and non-clinical levels of maternal anxiety and offspring anxiety (Lawrence et al. 2019). More specifically, from very early stages of infancy onwards, intergenerational links are observed between maternal anxiety and early temperament-based risk factors of childhood anxiety, with abundant research pointing towards the specific risk of prenatal maternal anxiety (for review see Korja et al. 2017) as well as postpartum maternal anxiety (Goodman et al. 2016) for behavioral and neurobiological aspects of infant NR (Davis et al. 2004; Reck et al. 2013) and temperamental fearful withdrawal during the first year of life (Reck et al. 2013; Van den Heuvel et al. 2015).

In light of evidenced intergenerational links between maternal anxiety (both prenatal and postnatal) and early temperamental risk factors of childhood anxiety, questions arise about the mechanisms that might underlie such links. Research reveals that while 50% of the variance in maternal anxiety and child temperamental fearful withdrawal is accounted for by shared genetic material (Pérez-Edgar and Fox 2018), the remaining 50% appears to be accounted for by additional non-genetic mechanisms (biological and/or behavioral). While biological mechanisms may be particularly pertinent for prenatal effects, behavioral mechanisms may be more relevant for postnatal effects. For instance, dysregulated glucocorticoids, which affect the fetus’ brain development, have been suggested to explain the link between prenatal maternal anxiety and offspring’s later anxiety (Davis and Sandman 2012). Postnatally, behavioral features of the caregiving environment have been identified as mechanisms of intergenerational transmission, such as modeling, where infants imitate mothers’ behavioral expressions of fear toward novel stimuli (Nimphy et al. 2023). Moreover, in older children, mounting evidence points towards caregiving mechanisms, such as harsh discipline, overprotection, and parental encouragement of avoidance, that play an important role in linking maternal anxiety to childhood temperamental fearful withdrawal (for review see, Rapee and Bayer 2018). These findings have informed interventions aimed at ameliorating the stability of temperamental fear in toddlers and children, with accumulating empirical evidence indicating the effectiveness of interventions that focus on parent-child interactions (for review see, Rapee and Bayer 2018). Nevertheless, whether perinatal caregiving mechanisms such as bonding with offspring might play similar roles in the early links between maternal perinatal anxiety and infants’ temperamental fear, remains largely unknown. Such information would be crucial for the targeting of early prevention mechanisms that could be deployed prior to the distinct expression of temperamental fearful withdrawal and its associated long-term risks for anxiety.

### Maternal bonding and infants’ temperamental risk

Maternal bonding represents one of the earliest processes of mothering, referring to the subjective emotional tie that a mother feels towards her infant, as expressed in warm and positive emotions, thoughts, and responses (Brockington et al. 2001; Le Bas et al. 2020). Individual differences exist in the emergence of bonding, and for some mothers, bonding is impaired. Impaired bonding may be reflected by increased maternal feelings of distance from the baby, regret of having the baby, becoming irritated by the baby, and not enjoying playing or cuddling with the baby (Brockington et al. 2001; Le Bas et al. 2020). A recent meta-analysis by O’Dea and colleagues (2023) reported that 3–22% of parents experience mild to moderate bonding impairments, according to self-report. These rates are elevated in mothers with postpartum depression (Gilden et al. 2020). Importantly, impaired maternal bonding, as described in this study, should be distinguished from the highly prevalent maternal experience of thoughts of infant-related harm (Lawrence et al. 2017). Approximately 70–100% of mothers experience unwanted, intrusive thoughts surrounding accidental or even intentional harm to their infant (Abramowitz et al. 2003; Fairbrother and Woody 2008). While the presence of these thoughts has been found to be elevated in the context of maternal anxiety, research demonstrates that such thoughts rarely translate into actual harmful behaviors (Fairbrother and Woody 2008; Lawrence et al. 2017). Individual differences in maternal bonding are observed from prenatal stages onward, in varying maternal emotions towards her fetus and attitudes towards pregnancy and the maternal role (Alhusen 2008).

Abundant literature suggests that maternal bonding plays a crucial role in fostering maternal co-regulation during the first months of life, which is supportive of infant’s emerging regulatory capacities (Bernier et al. 2010; Johnson 2013), which in turn are protective against risk for NR and temperamental fearful tendencies (Pérez-Edgar and Fox 2018). In addition, during pregnancy, maternal bonding might indirectly reduce risk for infants’ NR and temperamental fearful tendencies, through fostering postnatal bonding (Dubber et al. 2015), as well as directly reduce risk through lower levels of in utero exposure to stress-hormones (for review see, Weinstock 2008), alcohol and nicotine (Lindgren 2003). To date, several empirical studies have supported a link between early maternal postnatal bonding and infant NR (Nolvi et al. 2016) and temperamental fearful withdrawal (Parfitt et al. 2014; Takács et al. 2020). Nevertheless, the link between prenatal maternal bonding and infant NR and temperamental fearful withdrawal has not been previously examined, and such information would be of importance for prenatal preventive initiatives, allowing to be employed early on, before patterns of mother-infant interaction have crystalized (Kivijärvi et al. 2005; Spinrad and Stifter 2002).

### Maternal anxiety and maternal bonding

From a theoretical point of view, most scholars agree that maternal anxiety is likely to place mothers at increased risk for impaired bonding, due to overwhelming personal feelings and struggles which may interfere with mother-infant bonding and maternal support of her infant’s needs (Nolvi et al. 2016; Reck et al. 2013). Nevertheless, from an empirical point of view, the specific contribution of maternal anxiety to maternal bonding during infancy remains unclear (Göbel et al. 2018). Emerging evidence suggests negative associations between maternal anxiety and maternal bonding, both when assessed prenatally (Figueiredo and Costa 2009; Göbel et al. 2018) and postnatally (Dubber et al. 2015; Tolja et al. 2020). Nonetheless, some studies have indicated that these negative associations do not always hold when statistically controlling for demographic covariates or maternal comorbid symptoms such as maternal depression (Dubber et al. 2015; Tolja et al. 2020). In addition, we are aware of one study that reported significant associations in the opposite direction such that higher levels of maternal anxiety were associated with and higher levels of bonding (Edhborg et al. 2011). Importantly, the latter work was performed in a low SES sample in rural Bangladesh and cultural differences might be underlying these specific results.

### Maternal bonding as a mechanism underlying links between maternal anxiety, infant NR and infant fearful withdrawal

Studies reveal direct links between maternal anxiety and early maternal bonding impairments (Dubber et al. 2015; Figueiredo and Costa 2009; Göbel et al. 2018; Tolja et al. 2020), as well as direct links between early maternal bonding impairments and infant temperamental tendencies for NR and fearful withdrawal (Nolvi et al. 2016; Parfitt et al. 2014; Takács et al. 2020). Nonetheless, studies have not yet examined whether maternal bonding plays a mechanistic role in the early intergenerational transmission of maternal anxiety to infant NR and fearful withdrawal. In infancy, we are aware of one study that has assessed a similar research question, with results suggesting that impairments in maternal bonding at 3 months postpartum play a mechanistic role in the association between maternal symptoms of anxiety during the third trimester of pregnancy and associated risk for infant socio-emotional developmental assessed at one year (Le Bas et al. 2021). Infant socio-emotional developmental risk taps into broader capacities than those associated with temperament-based risk factors for anxiety, and whether the same indirect mechanism might underlie the early development of NR and temperamental fearful withdrawal throughout the first year of life remains unknown.

### Bi-directionality

Most of the previous studies revealing direct links between maternal anxiety, maternal bonding, and infant NR or temperamental fearful withdrawal have included cross-sectional designs which do not allow for pinpointing the predictive direction of associations. More specifically, largely based on previous work on the impact of child characteristics on family interactions, current developmental theories highlight the transactional nature of intergenerational transmission in the sense that children affect parents as much as parents affect children (Henderson et al. 2018). There is theoretical consensus that intergenerational processes are best conceptualized as back-and-forth influences between parents and children, and their relationship, over time (Henderson et al. 2018). This transactional nature has been empirically supported for associations between maternal anxiety, parental caregiving characteristics, and child anxiety (Ahmadzadeh et al. 2019; Henderson et al. 2018; see Fox et al. 2023 for a review). For example, it has been well established that parental anxiety impacts children’s later anxiety (Lawrence et al. 2019), and a growing body of research suggests that children’s fear and anxiety may also influence parents’ own anxiety (Buss et al. 2021; Fox et al. 2023). Moreover, research reveals bidirectional effects of toddler temperament and maternal behavior on both maternal and child anxiety (Buss et al. 2021). Nevertheless, research on transactional processes involving maternal anxiety, maternal bonding and infant reactivity or temperamental fearful withdrawal across the first year of life is scarce. The few studies that did examine this topic point towards bidirectional links between maternal bonding and maternal anxiety (Figueiredo and Costa 2009) as well as between maternal bonding and infant temperament (Takács et al. 2020).

### The current study

The current study is the first to empirically investigate a longitudinal predictive indirect pathway of risk between perinatal maternal anxiety, maternal bonding, and infant temperamental precursors of anxiety (infant NR and fearful withdrawal) from the prenatal period and across the first year of the infant’s life. Although previous work has identified patterns of intergenerational transmission of perinatal anxiety very early on, and within the first year of life, to date no study has examined whether the mechanism of early maternal perinatal bonding with her infant might underlie the indirect link between prenatal anxiety and early infant temperamental anxiety risk. Such results would inform the planning of prevention efforts emphasizing the potential benefit of targeting impaired maternal bonding, which may be relatively easily addressed and altered in clinical practice (Gilboa et al. 2018; Ji et al. 2005; Johnson 2013; Schlesinger et al., 2020; Simpson et al. 2003). Furthermore, such results would highlight the importance of early screening during pregnancy, which would allow for implementing prevention efforts early on and prior to the development of impaired bonding. Finally, identifying a target mechanism within the first year of life would highlight the possibility for preventing intergenerational transmission of risk prior to the onset of child anxiety.

To investigate the longitudinal pathway of risk, the current study employed a repeated measures study-design in which maternal general perinatal anxiety and bonding were measured prenatally (T1), one month postpartum (T2), four months postpartum (T3), and ten months postpartum (T4). In addition, infant NR and temperamental fearful tendencies were measured when they come online at four and ten months respectively (Pérez-Edgar and Fox 2018).

Based on previous literature, we hypothesized that maternal anxiety at T1 predicts maternal bonding at T2, which predicts infant NR at T3 and infant temperamental fearful withdrawal at T4 (hypothesis 1). Moreover, considering bidirectional associations, we hypothesized significant associations between higher levels of infant NR at T3 and lower levels of maternal bonding at T4, as well as associations between lower levels of maternal bonding at T1, T2, and T3 and higher levels of maternal anxiety at T2, T3, and T4, respectively (hypothesis 2).

## Methods

### Participants and procedure

Participants were recruited during the third trimester of pregnancy at a large community hospital in central Israel, where they were waiting for routine checkups. Exclusion criteria were pre-planned elective C-section, premature birth, multiple pregnancy, and known serious medical issues at time of recruitment. The current sample includes 216 mother-infant dyads. Mothers completed self-report questionnaires on anxiety and bonding at four time-points: T1) third trimester of pregnancy; T2) one month postpartum; T3) four months postpartum; T4) ten months postpartum. At T3 and T4, mothers also completed questionnaires on their infant’s temperament. In addition, at T3, a research assistant visited the participants at home and performed the infant reactivity paradigm. At T4, participants came to the laboratory, and temperamental fearful withdrawal was assessed by means of the Laboratory Temperament Assessment Battery (Lab-TAB) unpredictable toy procedure. From the T1 sample, 76% of dyads participated at T2, 56.0% at T3 and 43.1% at T4. The attrition rate was comparable to that of other studies with similar samples of new mothers and their infants (de Vente et al. 2011; Fox et al. 2015; Parfitt et al. 2014). At T1, mothers were aged between 21 and 44 years (*M* = 32.78, *SD* = 3.92) and had between 12 and 22 years of education. 59.7% experienced a first birth; and 48.1% of the infants were girls. 11.8% of the participants had a monthly household income below 1700 US Dollars; 25.0% between 1700 and 3300 US Dollars; 23.5% between 3300 and 5000 US Dollars; 22.1% between 5000 and 7000 US Dollars; and 17.6% above 7000 US Dollars. As such, the majority of our sample has a household income in line with or slightly above Israel’s average household income of 4500 US Dollars (Central Bureau of Statistics 2019). All participants were white, primarily middle-class families, which is reflective of the composition in the region (Central Bureau of Statistics 2019). Ethics approval was received from the authors’ IRB which works in line with the 1964 Helsinki declaration and its later amendments. Mothers provided written informed consent.

### Measures

**Maternal report.** Mothers reported on their *general anxiety* by means of the Trait Anxiety Inventory (Spielberger 1983) at T1 and T4, and by means of the State Anxiety Inventory (Spielberger 1983) at T2 and T3. The State Anxiety Inventory was included at T2 and T3 in order to capture changes in anxiety during the immediate postnatal transitory phase. The trait anxiety inventory asks participants to answer a series of questions, thereby indicating how they generally feel. Example items are “I feel nervous and restless”; “I feel satisfied with myself”; “I feel like a failure”; “I feel that difficulties are piling up so that I cannot overcome them”; “I make decisions easily”. The state anxiety inventory includes a series of questions for which participants are asked to indicate how they feel right now, at this very moment. Example items are “I feel calm”; “I feel tense”; “I feel at ease”; “I feel uncomfortable”; “I feel relaxed”; “I feel nervous”. Both Inventories of Trait Anxiety and State Anxiety have been used frequently in perinatal samples (Araji et al. 2020; Dennis et al. 2017). Cronbach’s Alphas were 0.88 (T1), 0.90 (T2), 0.92 (T3), and 0.90 (T4), indicating good to excellent internal consistency (Koo and Li 2016).

In addition, in the current study we controlled for *maternal depression*, on which mothers reported by means of the Edinburgh Postpartum Depression Scale (EPDS; Cox 1990; Cox et al. 1987) at T1, T2, and T3, and by means of the Beck Depression Inventory (BDI; Beck et al. 1961) at T4. EPDS was used at the first three timepoints due to its specific sensitivity to the perinatal period. Example items from the EPDS are, “I have blamed myself unnecessarily when things went wrong,” and “I have been so unhappy that I have been crying.” The BDI includes items such as, “I am disappointed in myself,” “I am less interested in other people or things than I was before,” and “I feel quite guilty most of the time.” In previous studies, both EPDS and BDI have been extensively used during the perinatal period, with EPDS being most often used during pre- and early postpartum periods, and BDI during later stages of the postpartum period (Cox 1990; Cox et al. 1987; Slomian et al., 2019). Cronbach’s Alphas were 0.84 (T1), 0.84 (T2), 0.82 (T3), and 0.79 (T4), suggesting good internal consistency (Koo and Li 2016).

Mothers reported on their *bonding towards their infant* by means of the Prenatal Attachment Inventory (PAI; Muller and Mercer 1993; T1) and the Postpartum Bonding Questionnaire (PBQ; Brockington et al. 2001; Brockington et al. 2006; T2, T3, and T4). The PBQ originally consisted of 25 items, however in line with previous studies we excluded two questions tapping into risk of infant abuse, due to poor psychometric properties (Brockington et al. 2006). Example items of the PAI are, “I get very excited when I think about the baby” and “I enjoy feeling the baby move” and example items of the PBQ are, “I feel happy when my baby smiles or laughs” and “My baby winds me up.” We reverse scored items referring to impairments in bonding (e.g., my baby irritates me), so that higher scores represent greater maternal bonding towards her infant. Cronbach’s alphas were 0.89 (T1), 0.76 (T2), 0.73 (T3), and 0.69 (T4), indicating moderate to good internal consistency (Koo and Li 2016).

Mothers reported on their infants’ *temperament-based risk factors* for anxiety by means of the negative affectivity scale (T3) and fear scale (T4) of the Infant Behavior Questionnaire – Revised (Gartstein and Rothbart 2003). The negative affectivity scale measures infants’ general disposition to experience negative emotions. Example items are “When tired, how often did your baby show distress?” and “How often did the baby seem angry (crying and fussing) when you left her/him in the crib?” The fear scale measures infant startle or distress to different naturally occurring fear-eliciting situations, such as novel stimuli or sudden changes in stimulation or environment. Example items are “How often during the last week did the baby startle at a sudden change in body position (e.g., when moved suddenly)?”; “When an unfamiliar person came to your home or apartment, how often did your baby cry when the visitor attempted to pick her/him up?” Cronbach’s Alphas were high at T3 (0.80) and T4 (0.82), indicating good internal consistency (Koo and Li 2016).

**Observation. ***Infant reactivity.* During the T3 home visit at age 4 months, infants underwent the infant reactivity paradigm, during which they were presented with a series of novel stimuli, both social (audio recordings of human voices) and non-social (mobiles), that became increasingly complex over time (for an elaborate description see Calkins et al. 1996; Fox et al. 2001). Infant behavior was videotaped and subsequently coded for level of negative affect (indexed by duration of fussing, fretting, and crying), and gross motor arousal (indexed by frequency and intensity of arm and leg movements). For each child, total continuous scores were calculated for negative affect and gross motor arousal by averaging its according standardized coded values. A final continuous reactivity score was calculated by averaging the negative affect and gross motor arousal scores. Coding work was divided between two experienced coders who were blind to the study hypotheses. 20% of the total sample was double coded in order to assess inter-coder reliability. Intraclass correlation coefficient (ICC) was 0.90 for negative affect and 0.74 for gross motor arousal codes, indicating moderate to excellent internal consistency (Koo and Li 2016). These reliability estimates are in line with previous work using this paradigm, demonstrating somewhat lower reliability estimates for motor arousal (*r* = 0.68) than for negative affect (*r* = 0.95; Fox et al. 2001).

*Temperamental fearful-withdrawal.* During the T4 laboratory visit at 10 months, infants underwent the unpredictable toy paradigm from the Lab-TAB, designed to elicit fear (for an elaborate description see Goldsmith and Rothbart 1999). For the two 5-seconds epochs of toy-bark, we behaviorally coded peak intensity of infant escape (0–3), infant vocal distress (0–5), and infant startle response (0–3). For each child, a total fearful withdrawal score was calculated by averaging the standardized scores for startle, escape, and distress vocalization. All videos were coded by the same experienced coder, and 20% of the sample was double coded by a second experienced coder. Coders were blind to the study hypotheses. Double coding yielded an overall Intraclass Correlation Coefficient (ICC) of 0.68, indicating moderate internal consistency (Koo and Li 2016), which is in line with Lab-TAB task reliability estimates in other studies (Calkins et al. 2002; Gagne et al. 2011).

### Data-analytic strategy

**Fig. 1:**
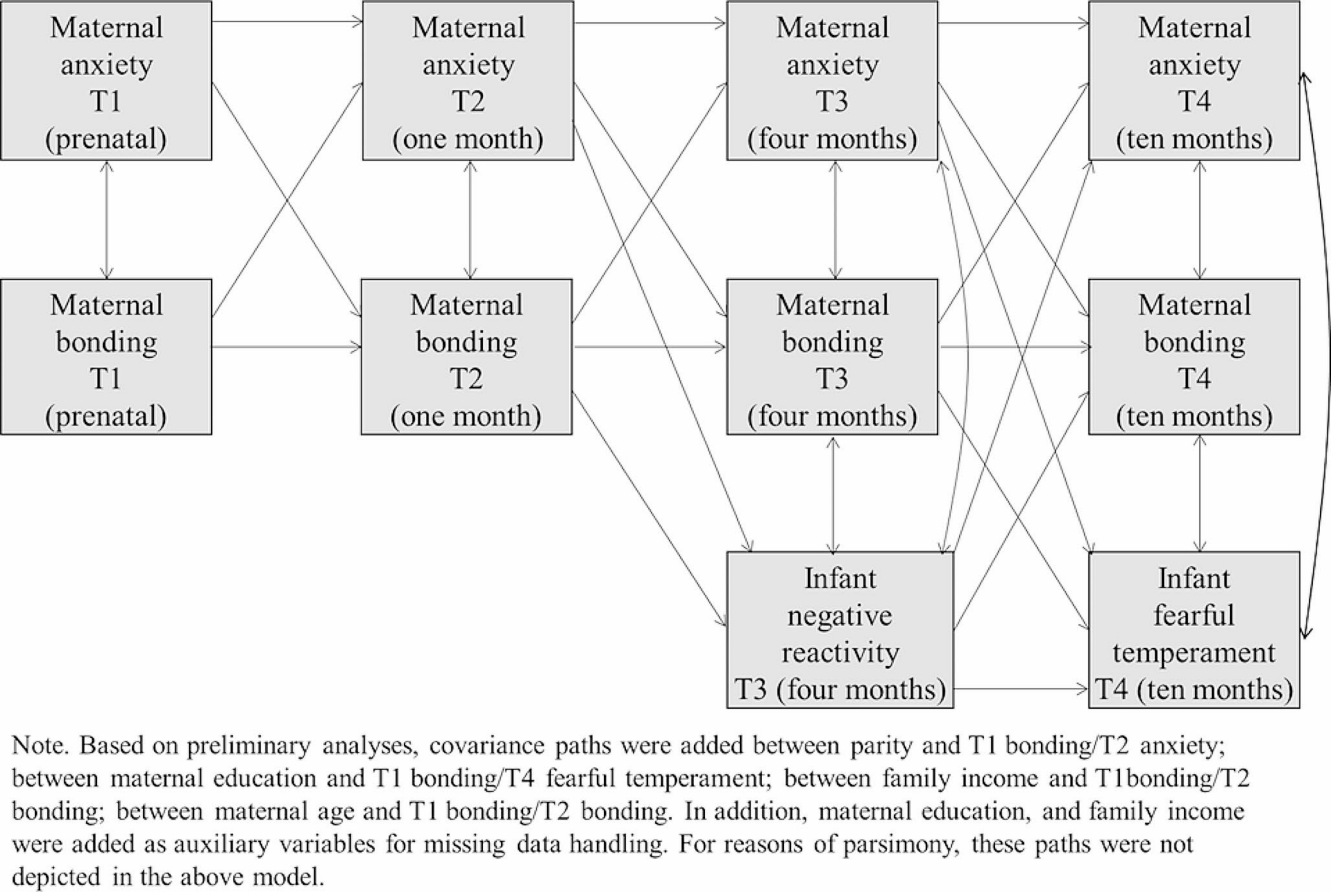
Full longitudinal cross-lagged model

All analyses were performed in Mplus version 8.8 (Muthén & Muthén, 1998–2017). Preliminary analyses assessed correlations between all study variables, and associations with potential covariates. Associations with potential covariates were assessed by means of independent sample t-tests (for covariates sex and parity) and correlations (for covariates maternal age, maternal years of education, family income, gestation week, and infant age at the various moments of data-collection). Main analyses were performed using cross-lagged longitudinal path modelling, and covariates that were significantly associated with a study variable were included in the model by regressing them on that specific study variable (for sex and parity, dummy scored variables were used). First, we tested the full hypothesized model (see Fig. 1). Model fit was assessed by means of the likelihoodratio test (χ^2^), Bentler comparative fit index (CFI), and root mean square estimation of association (RMSEA). Good model fit was indicated by a non-significant χ^2^, a RMSEA lower than 0.06 (> 0.1 suggests poor fit), and a CFI larger than 0.95. Model modifications were made based on modification indices (we added paths with a modification index > 10.00 and theoretical justification) and standard estimates of contribution to the model (we removed paths with standard estimates < 0.2). In order to assess whether the effects of maternal anxiety were not elicited by comorbid maternal depression, the final model was run again with the addition of covariances between maternal anxiety and maternal depression at each timepoint.

Missing data was handled by model estimation through Full Information Maximum Likelihood estimation (FIML), which is the preferred approach for avoiding biased statistical inferences (Schafer and Graham 2002). Moreover, FIML is shown to be reliable even for high percentages of missing data, without risk of loss of power or requirement of increased sample size (Schafer and Graham 2002). FIML can be used under the assumption of “missing data completely at random”. In the current study, this assumption will be tested by means of Little’s MCAR test, for which *p* > 0.05 indicates “missing data completely at random”. If Little’s MCAR test indicates that data is not missing completely at random, then associations will be calculated between the amount of missing data and demographic covariates (infant sex; infant age at data-collection; maternal age; maternal years of education; family income; gestation week at the time of prenatal assessment). In line with standard guidelines, demographic covariates that show significant associations with amount of missing data will be adopted as auxiliaries in the missing data handling process, such that the “missing at random” hypothesis can be assumed (Schafer and Graham 2002). Because in Mplus, the missing data auxiliary function cannot be employed in parallel to estimation of modification indices, auxiliaries will be added in the final stages of modelling. To avoid local solutions/maxima, the numbers of random starts and final stage optimalizations will be set to 5000 and 1000, and model results will be interpreted only on the condition that the best log-likelihood value is replicated (Hipp and Bauer 2006). Power analyses indicated that for the hypothesized model, a sample size of 187 is needed, given the conservative approach of an expected low effect size, power of 0.8, and alpha of 0.05.

### Transparency and openness

We report how we determined our sample size, all data exclusions (if any), all manipulations, and all measures in the study, and we follow JARS (Kazak 2018). Materials and analysis code for this study are available by emailing the corresponding author. All analyses were conducted using Mplus software version 8 (Muthén, & Muthén, 1998–2017). This study’s design and its analyses were not pre-registered.

## Results

Prior to model testing, we explored descriptive statistics and correlations for the observed and maternal reported temperament variables (Table 1). Both at T3 and T4 there were positive and significant correlations between observed and maternal reported variables and as such, composites were made which will be used in all further analyses. More specifically, for T3, we first z-scored all variables and then averaged maternal reported negative affectivity with the average of observed motor reactivity and observed negative affect reactivity. Likewise, for T4, we first z-scored all variables and then averaged maternal reported temperamental fearful withdrawal with the average of observed startle response, vocal distress, and escape.

**Table 1:** Descriptives and correlations for observed and mother reported temperament

		M	SD	Correlations					
				2	3	4	5	6	7
**Age Four Months**									
	**Observed reactivity**								
1	Gross motor reactivity	0.25	0.14	.29^**^	.23^*^	-.10	-.05	-.04	.16
2	Negative affect reactivity	0.22	0.29	1	.04	-.02	.05	.06	-.11
3	**Maternal report negative affectivity**	3.32	0.96		1	.19	.42^**^	.49^***^	.58^***^
**Age Ten Months**									
	**Observed temperamental fearful withdrawal**								
4	startle response	0.37	0.63			1	.54^***^	.59^***^	.30^**^
5	vocal distress	0.27	0.82				1	.74^***^	.40^**^
6	escape	0.19	0.50					1	.49^***^
7	**Maternal report temperamental fearful withdrawal**	2.69	1.20						1

^***^p < 0.001; ^**^p < 0.01; ^*^p < 0.05

Table 2 gives an overview of descriptive statistics and correlations for all study variables. Several significant associations emerged between higher levels of maternal anxiety and lower levels of maternal bonding. Specifically, higher levels of prenatal anxiety were associated with lower levels of concurrent prenatal bonding. Similarly, higher levels of postnatal anxiety were significantly associated with almost all measurements of postnatal bonding. In turn, lower levels of maternal bonding at T2 and T3 were significantly linked with increased levels of infant NR at T3, whereas higher levels of prenatal anxiety were significantly associated with higher levels of infant NR at T3. No associations emerged between any of the maternal anxiety or bonding measures and temperamental fearful withdrawal assessed at 10 months. However, increased infant NR at 4-months was significantly linked with increased infant temperamental fearful withdrawal at 10 months.

In addition, we explored associations between all study variables and potential covariates, by means of independent sample t-tests (for the covariates sex and parity) and correlations (for the covariates maternal age, maternal years of education, family income, gestation week, and infant age at the various moments of data-collection). Results showed that compared to multiparous mothers, primiparous mothers experienced higher levels of T1 bonding (*t*(210)=-3.86; *p* < 0.001) and higher levels of T2 anxiety (*t*(136)=-2.85; *p* = 0.005). More educated mothers reported lower levels of T1 maternal bonding (*r*=-0.25; *p* < 0.001), and had infants with higher levels of T4 temperamental fearful withdrawal (*r* = 0.22; *p* = 0.036). Higher family income and maternal age were both associated with less T1 maternal bonding (*r*=-0.26, *p* < 0.001; *r*=-0.18, *p* = 0.008) and less T2 maternal bonding (*r*=-0.21, *p* = 0.013; *r*=-0.17, *p* = 0.037). None of the study variables were significantly associated with infant sex, gestation week, or infant age at the various moments of data-collection (*p*’s > 0.082). In subsequent analyses, covariates were included based on the above identified significant associations.

**Table 2:** Descriptives and correlations for main study variables

		*n*	Min	Max	M	SD	2	3	4	5	6	7	8	9	10
Maternal Anxiety															
1	T1 (prenatal)	212	1.00	3.25	1.57	0.39	.40^***^	.41^***^	.73^***^	-.14^*^	-.39^***^	-.47^***^	-.24^*^	.26^*^	.02
2	T2 (postnatal)	138	1.00	3.55	1.46	0.41	**1**	.63^***^	.49^***^	-.14	-.49^***^	-.50^***^	-.29^*^	.09	-.08
3	T3 (postnatal)	82	1.00	2.95	1.36	0.41		**1**	.43^**^	-.01	-.34^**^	-.45^***^	-.26	.04	-.05
4	T4 (postnatal)	81	1.00	3.10	1.62	0.42			**1**	-.18	-.38^**^	-.47^***^	-.51^***^	.09	-.01
Maternal Bonding															
5	T1(prenatal)	212	1.39	4.00	3.02	0.54				**1**	.40^***^	.19^*^	.17	-.16	-.11
6	T2 (postnatal)	154	4.17	6.00	5.50	0.38					**1**	.72^***^	.54^***^	-.26^**^	.11
7	T3 (postnatal)	106	3.96	6.00	5.59	0.33						**1**	.54^***^	-.31^**^	-.10
8	T4 (postnatal)	62	4.42	6.00	5.59	0.32							**1**	-.15	.15
Temperamental Precursors of Childhood Anxiety															
9	T3 (Negative Reactivity)	111	-2.08	2.17	-0.07	0.80								**1**	.39^***^
10	T4 (temperamental fearful withdrawal)	90	-1.50	4.91	-0.07	0.85									**1**

^***^p < 0.001; ^**^p < 0.01; ^*^p < 0.05

T1 = end of the third trimester of pregnancy; T2 = one month postpartum; T3 = four months postpartum; T4 = ten months postpartum

Across all main study variables, Little’s MCAR was trend significant (*χ*^2^(244) = 274, *p* = 0.091), suggesting that data might not be missing “completely at random”. Follow-up t-tests and correlation analyses showed that more missing data was significantly associated with less years of maternal education (*r*=-0.219; *p* = 0.001), and lower family income (*r*=-0.138; *p* = 0.049). None of the other potential covariates were associated with amount of missing data (*p*’s > 0.060). As such, in line with standard guidelines, in what follows, maternal education, and family income, will be adopted as auxiliaries in the missing data handling process, such that the “missing at random” hypothesis can be assumed (Schafer and Graham 2002). Because in Mplus, the missing data auxiliary function cannot be employed in parallel to estimation of modification indices, auxiliaries were added in the final stages of modelling (see below).

**Fig. 2:**
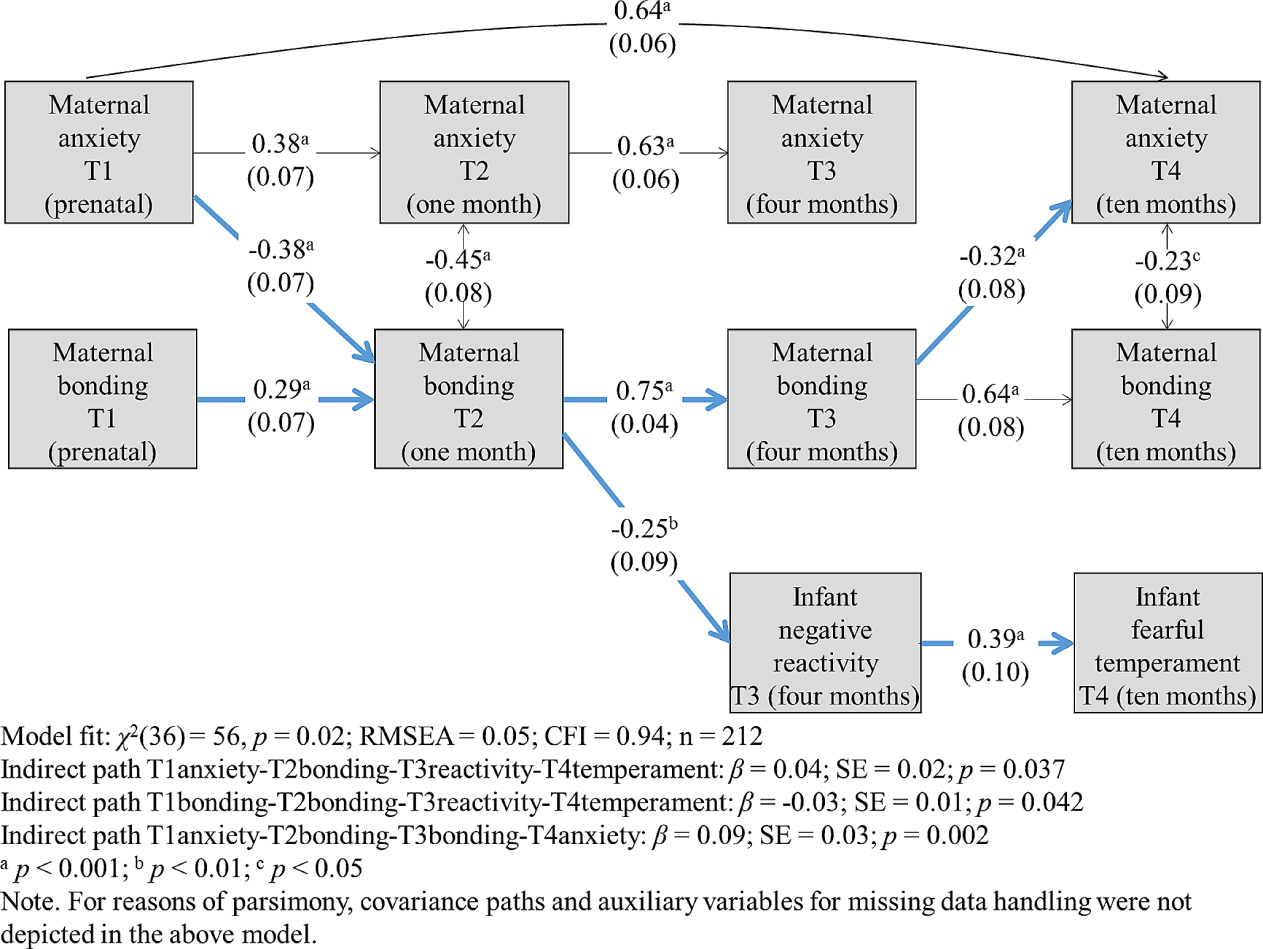
Final model

As a first modelling step, we fitted the full longitudinal cross-lagged model (see Fig. 1), which did not fit the data well (*χ*^2^(56) = 218, *p* < 0.001; RMSEA = 0.12; CFI = 0.66; *n* = 212). Based on model modification indices, we added a covariance path between T1 maternal anxiety and T4 maternal anxiety. Next, we removed all paths with standard estimates < 0.2 (all *p*’s > 0.05), and added auxiliary variables. The final model had a good fit (*χ*^2^(36) = 56, *p* = 0.02; RMSEA = 0.05; CFI = 0.94; *n* = 212), and is presented in Fig. 2. Importantly, the final model included two significant indirect predictive pathways to infant temperamental fearful withdrawal. The first indirect predictive pathway ran from prenatal maternal anxiety to maternal feelings of bonding towards her infant one month postpartum, to infant NR four months postpartum, and to infant temperamental fearful withdrawal ten months postpartum (*β*_indirect_ = 0.04, SE = 0.02, *p* = 0.037). The second indirect predictive path ran from prenatal maternal feelings of bonding towards her infant, to maternal feelings of bonding towards her infant one month postpartum, to infant NR four months postpartum, and to infant temperamental fearful withdrawal ten months postpartum (*β*_indirect_=-0.03, SE = 0.01, *p* = 0.042). In addition, a significant indirect predictive pathway was observed from prenatal maternal anxiety, to maternal feelings of bonding towards her infant one month postpartum, to feelings of bonding towards her infant at four months postpartum, and to maternal anxiety at ten months postpartum (*β*_indirect_ = 0.09, SE = 0.03, *p* = 0.002). The total explained variance in infant NR was *R*^2^ = 0.06 and the total explained variance in infant temperamental fearful withdrawal was *R*^2^ = 0.15. Importantly, this final model had a great fit when run for those participants with no missing data only (*χ*^2^(28) = 26, *p* = 0.56; RMSEA = 0.00; CFI = 1.00; *n* = 78). In order to assess whether the effects of maternal anxiety were not elicited by comorbid maternal depression, the final model was run again with the addition of maternal depression at each timepoint, and specifying covariances between maternal anxiety and maternal depression at each timepoint. This model had good fit (*χ*^2^(69) = 107, *p* = 0.002; RMSEA = 0.050; CFI = 0.940) and all above specified significant indirect pathways between maternal anxiety, bonding, and infant temperament-based temperamental precursors of childhood anxiety, remained.

## Discussion

Past research has indicated strong intergenerational links between maternal and offspring anxiety (Lawrence et al. 2019). More specifically, precursors of such links can be observed from early stages of child development onward, in the association between perinatal maternal anxiety and early temperamental precursors of later childhood anxiety - infant NR and temperamental fearful withdrawal during the first year of life (Davis et al. 2004; Reck et al. 2013; Van den Heuvel et al. 2015). The goal of the current study was to advance our understanding about potential environmental mechanisms that might be underlying these early intergenerational associations of anxiety. More specifically, we investigated the relevance of maternal bonding to her infant, one of the earliest caregiving processes developing during pregnancy. Using a longitudinal repeated measures design with four measurement points, from pregnancy to ten months postpartum, the current study was the first to investigate whether maternal anxiety places mothers at risk for impaired maternal bonding, which in turn predicts increased infant NR and temperamental fearful withdrawal.

Results confirmed our first hypothesis, by showing an indirect pathway from prenatal maternal anxiety, to infant NR at four months, which in turn predicted temperamental fearful withdrawal at ten months, through impaired maternal bonding at one month. Various processes may explain these observed links from prenatal maternal anxiety to infant NR through impaired maternal bonding. First, psychological processes are likely to play an important role. Mothers experiencing anxiety in the perinatal period may be preoccupied with personal struggles and therefore find it challenging to fully engage in nurturing a new relationship. Moreover, they may be anxious about bonding to a new individual due to fear of rejection or loss. In turn, these dynamics may disrupt co-regulatory practices, which are crucial for mitigating infant temperamental fear (Bernier et al. 2010; Johnson 2013). However, other processes, such as biological mechanisms, may also be important. For example, bonding emotions, cognitions, and behaviors are known to stimulate increased levels of the neuronal nonapeptide oxytocin (Hardin et al. 2020), which in turn mitigate infant NR and temperamental fearful withdrawal (Kajanoja et al. 2022). Our finding that caregiving processes of bonding may play a mechanistic role in the intergenerational link between maternal anxiety and infant NR, relates to previous research indicating associations between maternal anxiety and maternal bonding towards her infant (Dubber et al. 2015; Edhborg et al. 2011; Figueiredo and Costa 2009; Göbel et al. 2018), as well as between maternal bonding and infant NR and temperamental fearful withdrawal (Nolvi et al. 2016; Parfitt et al. 2014). Nevertheless, the current study was first to identify a longitudinal mechanistic pathway of risk. Such result has important implications for clinical practice as it suggests that the intergenerational risk of maternal anxiety to infant NR might be addressed by early prevention and intervention programs aimed at fostering early maternal bonding.

More specifically, the current findings indicate that such prevention and intervention initiatives might be directed at fostering both prenatal and postnatal bonding in mothers with anxiety, due to their predictive links with higher levels of later bonding, and lower levels of later infant NR, respectively. The importance of pre- and postnatal bonding for addressing the early intergenerational link between maternal and offspring anxiety is particularly promising, given the susceptibility of maternal bonding to relatively straightforward intervention techniques in normative populations, such as skin-to-skin contact and breastfeeding (Johnson 2013), ultrasound during pregnancy (Ji et al. 2005), and ultrasound during labor (Gilboa et al. 2018; Schlesinger et al., 2020). Indeed, previous work has suggested that bonding interventions have a particularly high success rate when provided during the perinatal period (Simpson et al. 2003). During the perinatal period, alterations in environment, hormones, and roles foster a unique window for potential change, even against the background of risk due to maternal anxiety (Simpson et al. 2003).

Few previous studies have investigated the mechanistic role of other caregiving processes in underlying the early intergenerational link between maternal anxiety and infant NR and temperamental fearful withdrawal. De Vente et al. (2011) did not identify an indirect pathway of risk between self-reported prenatal maternal anxiety, self-reported maternal fear for negative child evaluation at 4 months, and parent-reported infant fear at one year postpartum. Kaplan and colleagues (2008) did not identify an indirect path between maternal self-reported anxiety during pregnancy, observed maternal sensitivity at four months postpartum, and parent-reported infant fear at four months. Discrepancies between the results of our study and the null results of these previous studies can be explained by the way in which we measured infant NR and withdrawal tendencies. More specifically, a particular strength of our study was that we included a combination of parent report and direct observation (Rothbart and Bates 2006; Stifter et al. 2008). In addition, the discrepancy might be due to the fact that previous studies included only affective or behavioral aspects of parenting, whereas the current study encompassed both dimensions through bonding. In the scope of our current study, the choice for bonding was crucial, given that it comes online early, from pregnancy onwards. Nevertheless, future research may assess additional parenting dimensions, at the respective post-natal timepoints, and examine their relative contribution.

Our second hypothesis concerning bidirectionality of effects was partially confirmed. Specifically, we hypothesized that in addition to the associations described above, we would also observe associations between bonding impairments at earlier timepoints and maternal anxiety at later timepoints, as well as between infant temperament at earlier timepoints and maternal bonding impairments at later timepoints. In line with this hypothesis, impaired maternal bonding at T3 was associated with maternal anxiety at T4, however, contrary to our hypothesis, infant NR at T3 was not associated with impaired maternal bonding at T4. Concerning the latter non-significant result, scholars have suggested that early indicators of infant temperament may be observed from pregnancy onwards. Future research might include these measures to examine bidirectional links between child temperamental precursors of anxiety and maternal bonding prior to the age of four months. Overall, the partially confirmed bidirectional effects indicate that the targeting of bonding impairments in clinical practice may not only mitigate the intergenerational transmission of anxiety but also trigger cascading reciprocal effects leading to decreased maternal anxiety.

The present findings should be viewed in light of several limitations. First, the current study employed only self-report to operationalize maternal anxiety and bonding. Furthermore, only general perinatal anxiety was assessed, rather than specific perinatal anxieties related to pregnancy, childbirth, or motherhood. Though broad measures of anxiety, such as the STAI, are often used with perinatal samples and yield important findings (Araji et al. 2020; Dennis et al. 2017), other studies have utilized specific measures of anxiety, such as the Pregnancy-Related Anxiety Questionnaire-Revised (PRAQ-R; Huiznik et al., 2004). Both the STAI and the PRAQ have been found to relate to maternal bonding (Dubber et al. 2015), but future studies may examine whether general and specific anxieties in the perinatal period differentially relate to later reactivity and temperament in infants and whether maternal bonding similarly underlies these differential links.

Moreover, to further shed light on the links between maternal anxiety and bonding, future research may specifically wish to assess the normative intrusive and unwanted thoughts of infant-related harm, which are extremely common in the perinatal period (Abramowitz et al. 2003; Fairbrother and Woody 2008) and particularly enhanced in the context of maternal perinatal anxiety (Lawrence et al. 2017). Importantly, while research indicates that such thoughts rarely translate into actual harmful behavior, they elicit intense distress in mothers due to maternal fear of causing unwanted infant harm. As such, harm-related thoughts are thought to play an important role in maintaining maternal anxiety on the one hand and enhancing impaired bonding on the other hand (given maternal avoidance of infant due to maternal fear of causing infant harm; Lawrence et al. 2017). Future research including the specific assessment of infant harm thoughts may shed light on the planning of preventive interventions, perhaps highlighting the importance of normalizing such thoughts for reducing maternal anxiety and reducing maternal avoidance of the infant and associated risk for impaired bonding.

An additional weakness of the current study is that our sample included mainly middle- and high-income participants. Although this controls for potentially biasing factors related to socioeconomic status, a disadvantage is that the results might not generalize to other families. It should also be noted that our model predicted a modest amount of variance in infant NR (*R*^2^ = 0.061) and temperamental fearful withdrawal (*R*^2^ = 0.117), and effect sizes might increase when other potential predictors are taken into account such as paternal bonding. Finally, although the attrition rates in our study are comparable to those of other studies with similar samples of new mothers and their infants (de Vente et al. 2011; Fox et al. 2015; Parfitt et al. 2014), and although we aimed at reducing the impact of missing data by post-hoc statistical techniques, systematic bias might still have occurred.

Future research may assess the potential causal role that bonding plays in attenuating risk for infant NR and temperamental fearful withdrawal. Causality may be checked through intervention studies, in which mothers are randomly assigned to pre- or postnatal bonding initiatives versus care as usual. Comparing results for pre- and postnatal interventions may provide insight into sensitive periods during which bonding might reduce risk for infant NR and temperamental fearful withdrawal, or for the intergenerational transmission of anxiety. Interventions may be performed in both general population and clinical anxiety samples to understand whether the same mechanisms work for both groups. Moreover, further research should include fathers, as well as longer follow-up periods to investigate the long-term impact of early bonding.

Strengths of the current study include its four-wave longitudinal design with rapid follow-ups during the transitionary period of pregnancy and the first year of life. Additional strengths were our large sample size and the inclusion of both self-report and observation to assess infant NR and temperamental fearful withdrawal. In sum, the current study allowed to identify early maternal processes that potentially underlie the intergenerational transmission of anxiety, returning crucial implications for early intervention.

### Clinical implications

The current study identified an early interpersonal mechanism involved in the intergenerational transmission of maternal anxiety, i.e., maternal perinatal bonding. Findings inform the planning of prevention efforts during the perinatal period pointing towards several windows of opportunity for the implementation of preventive strategies from the end of pregnancy and across the first year of life. Results emphasize the potential benefit of targeting impaired maternal bonding both prenatally and postnatally to mitigate risk of intergenerational transmission of anxiety. These findings are particularly relevant for translation into practice given previous research indicating that early maternal bonding is relatively easy to foster in clinical practice (Gilboa et al. 2018; Ji et al. 2005; Johnson 2013; Schlesinger et al., 2020; Simpson et al. 2003). Furthermore, results highlight the importance of early screening of both maternal anxiety and maternal bonding during pregnancy and immediately postpartum. Identification of early risk would allow for implementing prevention efforts early on to prevent intergenerational transmission of maternal anxiety prior to the onset of child anxiety, and possibly prior to the onset of infant temperamental risk for anxiety. Taken together, the current study highlights the clinical importance of fostering maternal bonding toward her infant when encountering pregnant mothers experiencing anxiety and broadens the scope of clinical practice to include maternal bonding as an important factor for mitigating risk of intergenerational transmission of anxiety.
